# What is the appropriate control arm when testing usefulness of mobile stroke units in improving stroke outcomes?

**DOI:** 10.1177/15910199211011865

**Published:** 2021-04-19

**Authors:** Mayank Goyal, Rosalie McDonough, Johanna Maria Ospel

**Affiliations:** 1 Calgary Stroke Program, Department of Clinical Neurosciences, University of Calgary, Calgary, Canada; 2 Department of Radiology, University of Calgary, Calgary, Canada; 3 Department of Neuroradiology, University Medical Center Hamburg-Eppendorf, Hamburg, Germany; 4 Department of Radiology, University Hospital Basel, University of Basel, Basel, Switzerland

Recently, the first large, prospective study investigating the impact of mobile stroke units (MSU) for pre-hospital triage on patient outcomes in acute ischemic stroke was published.^1^ The thoughtfully designed and well-executed study by Ebinger et al.^1^ showed a significant benefit when MSU were used. When reading these results, one feels obliged to ask: what are the reasons for the improved outcomes in patients for whom a MSU was dispatched? Three possible answers come to mind:

1. Increased appropriate utilization of and faster treatment with intravenous alteplase.^2,3^ In patients with MSU dispatch, 60% received intravenous alteplase with a median dispatch-to-needle time of 50min, while in patients without MSU dispatch, only 48% received alteplase, with a median dispatch-to-needle time of 70min.
2. Increased expertise at the pre-hospital stage. MSUs were dispatched with an on-board neurologist, while conventional ambulances were dispatched with an emergency physician only in case of impaired consciousness or life-threatening conditions.
3. Enthusiasm of the MSU team members, who may feel more motivated than conventional ambulance staff by their pioneering role in changing acute stroke treatment.

Importantly, a novel stroke management strategy will only get accepted by healthcare providers if the healthcare system can afford it. The annual costs of operating an MSU in the United States for example have been estimated to be as high as 1.2 million USD (which does not account for the initial set-up cost of anywhere between 600.000 to 1 million USD),^4^ while a stroke neurologist’s salary is on average much lower, at 250.000 USD.^5^ We wonder if an “expert champion”. i.e., a physician taking the lead and guiding through the entire patient’s journey to the point of alteplase administration, would have the same impact but at a lower cost and easier implementation. Although an on-site champion would be preferable, telestroke networks could in theory be used for remote expert advice. The expert champion would:

1. Provide a higher level of expertise and targeted information collection during the 911-call, resulting in better decision making, more appropriate and faster allocation of resources.
2. Provide support to the paramedic team in the field through technologies such as video conferencing for clinical examination and other upcoming tools to detect LVO. This could substantially reduce on-site times and improve triage decisions.
3. Preparing the receiving hospital to reduce door-to-needle times by streamlining in-hospital workflows. The champion could inform the receiving hospital about the patient’s relevant medical history and medication and alteplase-eligibility status en route, prior to arrival of the ambulance. Upon arrival, the champion could direct the patient straight to the CT/MRI scanner, i.e., bypassing the emergency department, and, in close cooperation with the local team, immediately provide expertise for image interpretation and alteplase administration.

There are obvious advantages and disadvantages to both the MSU and the expert champion strategy. One clear advantage of MSUs is the option to administer IV alteplase prior to hospital admission, after a hemorrhage has been ruled out on CT. However, recent trials such as DIRECT-MT,^6^ DEVT,^7^ SKIP^8^ and MR CLEAN NO IV (results presented on March 18 at the international stroke conference) have raised the possibility that there is only little or no added benefit of IV alteplase in large vessel occlusion stroke patients who are undergoing timely EVT, although the non-inferiority margin in the latter two trials was not met. It is also important to note that these results only apply to a minority of patients, since on a population level, most stroke patients will not have a large vessel occlusion. The optimal management strategy will most likely depend on several factors, including available expertise, local geography and infrastructure. Clearly, further research that compares both strategies in different geographical settings is needed. However, we do believe that MSUs are generally very expensive and unlikely to become widely available in the near future. An expert who champions the patient’s entire journey from 911 call to alteplase administration could potentially have the same impact and, in our opinion, would serve as the appropriate control group and benchmark for a future MSU trial.
